# Comparative study of the chemical composition and antifungal activity of commercial brown seaweed extracts

**DOI:** 10.3389/fpls.2022.1017925

**Published:** 2022-12-13

**Authors:** Silvia Valverde, Paul Luis Williams, Begoña Mayans, Juan J. Lucena, Lourdes Hernández-Apaolaza

**Affiliations:** Department of Agricultural Chemistry and Food Science, Universidad Autónoma de Madrid, Madrid, Spain

**Keywords:** brown seaweed extracts, biostimulants, chemical composition, antifungal activity, chemometrics

## Abstract

**Introduction:**

A sustainable agriculture and the great increase in consumers of organic products in the last years make the use of natural products one of the main challenges of modern agriculture. This is the reason that the use of products based on seaweed extracts has increased exponentially, specifically brown seaweeds, including Ascophyllum nodosum and Ecklonia maxima.

**Methods:**

In this study, the chemical composition of 20 commercial seaweed extract products used as biostimulants and their antifungal activity against two common postharvest pathogens (Botrytis cinerea and Penicillium digitatum) from fruits were evaluated. Data were processed using chemometric techniques based on linear and non-linear models.

**Results and discussion:**

The results showed that the algae species and the percentage of seaweed had a significant effect on the final composition of the products. In addition, great disparity was observed between formulations with similar labeling and antifungal effect of most of the analyzed products against some of the tested pathogens. These findings indicate the need for further research.

## 1 Introduction

The great challenge of food production is to use sustainable practices that increase the harvestable yield and quality of crops with minimal impact on the environment. It is well-known that chemical fertilizers have led to the deterioration of agricultural systems (Biau et al., 2012). In recent years, environment-friendly and economic alternatives have been sought that can reduce the use of traditional fertilizers (Trivedi et al., 2018), such as seaweed extracts (SWEs). Recently, a growing interest has been observed for the use of this type of products because they are natural and biostimulant substances that improve the crop growth and quality of fruits (Tabbara et al., 2018). Despite the fact that SWEs are traditionally used as biostimulants, supplementary fertilizers, or soil quality improvers, their use has also been reported as animal feed supplement, human nutritional supplement, and cosmetic products (Pereira et al., 2020).

The most widely used macroalgae in agriculture are brown seaweeds, including *Ascophyllum nodosum* (*AN*), *Ecklonia maxima (EM)*, *Fucus vesiculosus*, *Laminaria digitata*, *Sargassum* spp., and *Turbinarias* spp. (Khan et al., 2009), being the most commercialized extracts of the first two species. Their biochemical composition depends on their location and the conditions of the place where they grow. In this way, the content of active ingredients will be modified between each species and within the same species in relation to the availability of nutrients, light, salinity, depth, presence of organic contaminants, or content of heavy metals (Kumar and Sharma, 2021) with potential human risk associated, among others. *A. nodosum* grows on the coasts of the North Atlantic, mainly on the northwestern coast of Europe and North America. Unlike other species of brown algae, it supports periods of marine immersion and periods of exposure to the elements, depending on the tidal cycle. This fact of physiological adaptation provides a characteristic biochemical composition that is very useful for their use in agriculture (Hurtado et al., 2021). Meanwhile, *E. maxima*, an endemic South African seaweed, always grows submerged in water and did not emerge at times of low tide; thus, it is considered a potential source of beneficial bioactive compounds. After seaweed recollection, the active ingredients contained in algae are extracted from the cells and transferred to a liquid medium to obtain a commercial product using different extraction processes. Depending on the process used, the final composition of the product can be significantly altered (Povero et al., 2016). The most widespread extraction processes are traditional extraction based on the use of chemical agents and cold extraction. Chemical extraction uses strong acids or alkalis (KOH), and the product obtained is dark due to the high oxidation of its active components leading to denaturation of active ingredients that results in a drastic loss of properties (Calvo et al., 2014). Cold extraction has gained popularity because algae are subjected to a micronized process under high pressure without loss of active ingredients and pH is kept at its physiological level of 4.5 (Crampon et al., 2011). These products have a characteristic marine aroma and color that ranges from green to light brown.

SWEs contain a multitude of active components (Khan et al., 2009), although it is considered that the following are the main active ingredients: alginic acid, which is present in the cell walls and confers flexibility and adaptation to stress phenomena; mannitol, which blocks reactive species and prevents metabolic damage; reserve polysaccharides (laminarin and fucoidan) that are not found in terrestrial plants; polyphenols and tannins with high antioxidant power, which stabilizes and strengthens cell walls against pathogen attacks and minerals, among others. All these components have a synergistic effect in the crops, providing a strong root system, promoting plant growth, and improving leaf development and flowering (Mukherjee and Patel, 2019). In addition, the quality of the fruit and vegetables has been reported in numerous studies, not only providing physiological and nutritional benefits but also improving the organoleptic properties by the consumer (Kocira et al., 2018; Meng et al., 2022). The antifungal activity of SWE has also been studied in several research as preventive treatment to postharvest pathogens (De Corato et al., 2017; Lahbib et al., 2022). This is because marine organisms have developed an antimicrobial defense strategy based on the production of bioactive metabolites (Cortés et al., 2014) with antioxidant character.

According to the growing organic production expansion and efforts to reduce the use of chemical fungicides and fertilizers, SWEs may play a greater role in crop protection than was previously realized. In fact, many products containing SWEs are commercially available for application to various horticultural crops under different trade names. In most of the products, there is no homogeneity in the labeling, and a detailed description of the composition is not usual, making it difficult to understand its mode of action and its influence on the plant growth, fruit quality, and antifungal activity.

Hence, this study was conducted to investigate the chemical composition and *in vitro* antifungal assays against two common pathogens on four different fresh fruits of 20 representative commercial seaweed extracts and to further treat data by multivariate statistical analysis. To the authors’ knowledge, this is the first time that such a detailed study of several brown seaweed extracts has been carried out. The contribution of this study is expected to provide a reference for the knowledge of these products and the ability of chemometrics multivariate methods to know the production factors that influence their composition and their relationship with antifungal activity.

## 2 Materials and methods

### 2.1 Samples

Several representative seaweed commercial products were selected in this study according to the species (*A. nodosum* and *E. maxima*), method of extraction (basic media-KOH, acid media, or cold), pH (from 3.20 to 10.8), the way in which they are marketed—liquid products (LP, n=16) or solid products (SP, n=4)—and percentage of algae in the product—low (10%–30%) and high (100%). Products with high percentage (100%) are those that consist only of algae extract, while those that have low percentage are mixtures of algae extracts with other components such as amino acids or sugars, among others. The percentage indicates the amount of seaweed in the product and is usually specified on the labeling. All the samples were kindly provided by different fertilizer companies (n=18) and by Laboratorio Arbitral (Spain’s Ministry of Agriculture, Fisheries and Food; MAPA) (n=2). **Table 1** summarizes the main characteristics of the seaweed products selected in this study.

**Table 1:** Description of the seaweed products selected for this study according to criteria described in *Section 2.1*.

Sample code	Formulated	Species	Algae percentage (%)	Extraction method	pH ± SD	EC (mS cm^-1^) ± SD
1	Liquid	AN	Low (25)	Cold	4.59 ± 0.01	5.34 ± 0.07
2		AN	Low (27)	Cold	4.26 ± 0.02	8.71 ± 0.1
3		AN	High (100)	Acid	3.35 ± 0.01	3.75 ± 0.02
4		AN	High (100)	Cold	4.40 ± 0.02	8.04 ± 0.09
5		AN	Low (27)	Cold	5.33 ± 0.03	20.1 ± 0.1
6		EM	Low (16)	Cold	5.20 ± 0.02	2.96 ± 0.01
7		EM	Low (15)	Cold	4.50 ± 0.01	2.17 ± 0.01
8		AN	Low (27)	KOH	10.0 ± 0.01	42.7 ± 0.2
9		AN	Low (30)	Acid	3.81 ± 0.01	10.2 ± 0.09
10		EM	Low (30)	Acid	3.79 ± 0.02	14.9 ± 0.1
11		AN	High (100)	Cold	4.70 ± 0.01	11.5 ± 0.09
12		AN	Low (20)	Cold	4.46 ± 0.01	8.33 ± 0.07
13		EM	High (100)	Cold	4.42 ± 0.01	13.1 ± 0.09
14		AN	High (100)	Cold	4.82 ± 0.02	9.01 ± 0.07
15		AN	High (100)	Cold	4.76 ± 0.01	5.55 ± 0.02
16		AN	High (100)	KOH	8.50 ± 0.01	13.2 ± 0.1
17	Solid	AN	High (100)	KOH	9.82 ± 0.03	12.1 ± 0.1
18		AN	High (100)	KOH	10.6 ± 0.02	20.4 ± 0.1
19		AN	High (100)	KOH	10.3 ± 0.03	20.2 ± 0.1
20		AN	High (100)	KOH	11.0 ± 0.03	21.0 ± 0.1

### 2.2 Sample preparation

Formulated liquid samples were previously freeze-dried and then pulverized with a mortar for all tests except for polysaccharides content (alginic acid, mannitol, and laminarin) and antifungal *in vitro* assays. Solid samples were previously homogenized with a mortar. All measurements were performed in triplicate.

#### 2.2.1 Determination of chemical composition

##### 2.2.1.1 Polysaccharides content

To estimate the alginic acid (AA), mannitol (Man-ol, polyol), and laminarin (Lam) content, the method of Valverde et al. (2022) was used. Briefly, 40 mg of liquid product (LP) or 20 mg of solid product (SP) was diluted in 100 ml (LP) or 250 ml (SP) of ultrapure water. For SP, a previous step was necessary based on crushing with a grind and passing it through a 40-mesh screen to obtain a homogenous sample. Then, the mixture was shaken in a vortex for 1 min and centrifuged for 5 min at 3,000 rpm. The samples were filtered through 0.45-µm nylon filter before analysis by high-performance liquid chromatography refractive index detector (HPLC-RID) system (1260 Infinity model Agilent Technologies, Waldbronn, Germany). A Bio-Rad Aminex HPX-87 H column (300 × 7.8 mm, 9 µm) was used, protected by a guard column from Phenomenex (Torrance, CA, USA). Analysis conditions were set as follows: the mobile phase was a 0.05% acetic acid, the flow rate was 0.5 ml/min, the injection volume was 50 µl, the column temperature was set at 65°C, and the temperature of the refractive index detector was at 50°C in positive polarity mode. Quantification was achieved using glycerol as internal standard (IS) calibration, employing glycerol in order to reduce analysis error (n=6) and fluctuations on the signal.

##### 2.2.1.2 Sugar content

Sugar analysis (arabinose, fucose, glucose, glucuronic acid, rhamnose, sucrose, and xylose) was carried out using the reported methods with slight modifications (Sluiter et al., 2012; Manns et al., 2014; Badmus et al., 2019): A total of 150 mg of the sample was hydrolysed with 200 µl of sulphuric acid 72% w/w at 30°C for 1 h; the reaction mixture was then diluted with 3.6 ml of ultrapure water reacting for 40 min at 100°C. After cooling, samples were centrifuged for 10 min at 5,000 rpm; ultrapure water was added, taking the supernatant up to 5 ml, and it was filtered through a 0.45-µm nylon filter before analysis by HPLC-RID system. A Bio-Rad Aminex HPX-87 H column (300 × 7.8 mm, 9 µm) was selected with 4 mM H_2_SO_4_ solution as the mobile phase at a flow rate of 0.6 ml/min; the injection volume was 50 µl, and the column temperature was set at 60°C.

##### 2.2.1.3 Mineral content

Mineral profiling was obtained through digestion of 50 mg of powdered seaweed weighed into an Erlenmeyer flask and addition of 5 ml of concentrated trace metal grade nitric acid (67% assay) and 5 ml of hydrogen peroxide (30% assay). The mixture was left to react for an hour at room temperature and then heated at 80°C for 30 min with a plate digestion. After cooling, samples were filtered and made up to 25 ml with Milli-Q water. The certified reference material (CRM) NCS DC73350 (leaves of poplar) was prepared in the same manner to validate the accuracy of the analysis. The samples were analyzed by inductively coupled plasma–optical emission spectrometry (ICP-OES) (iCAP-PRO XDuo Spectrometer, Thermo Scientific, UK). Fourteen minerals were measured: calcium (Ca), magnesium (Mg), potassium (K), aluminium (Al), phosphorous (P), iron (Fe), copper (Cu), manganese (Mn), zinc (Zn), nickel (Ni), arsenic (As), cadmium (Cd), chromium (Cr), and lead (Pb). The optimal instrumental conditions of ICP-OES, along with the selected wavelengths and IS, are presented in **Supplementary Table S1**. In order to avoid ion signal fluctuations caused by the matrix, a diluted internal standard (IS) solution (10mg/L of ^89^Y) was used. This solution was distributed in all solutions (blank, standard solutions, and unknown samples) using a second channel of the peristaltic pump.

##### 2.2.1.4 Hormonal composition

Plant hormones (abscisic acid, indole-3-acetic acid, indole butyric acid, gibberellic acid, and salicylic acid) were analyzed using reported validated analytical methodologies (Jiang et al., 2006; Górka and Wieczorek, 2017). Briefly, 50 mg of powdered samples were extracted with 0.5 ml methanol 70% (v/v) and remained overnight at 4°C. The supernatant was concentrated under N_2_ stream and dissolved in 1 ml Na_2_HPO_4_ at pH 9.0. The solution was extracted three times with ethyl acetate and adjusted to pH=2.5. Finally, the organic phases were dried with a gentle stream of N_2_, dissolved in 1 ml of methanol 70% (v/v), and filtered through 0.45-µm nylon filter before analysis by HPLC with a diode-array detector (DAD) (HPLC-DAD) system. Chromatographic conditions were set as follows: analytical column Luna C_18_ (2) (150 × 4.6 mm, 3 µm), mobile phase consisted of (A) acetonitrile (0.1% formic acid) and (B) 0.1% formic acid in gradient elution mode; 0–10 min A:B, 25:75 (v/v); 11–17 min A:B, 50:50 (v/v); and 18–30 min A:B, 25:75 (v/v). Flow rate was 0.5 ml/min, injection volume was 10 µl, and the temperature of the column was maintained at 25°C. Detection was carried out at a wavelength of λ=214 nm.

##### 2.2.1.5 Total phenol content

Methanolic SWE were obtained according to Badmus et al. (2019). Briefly, 250 mg of SWE and 5 ml of methanol was sonicated for 30 min and shaken for 4 h in an orbital shaker. Then, extracts were centrifuged for 10 min at 2,500 rpm. To estimate the total phenolic content (TPC), the modified Folin–Ciocalteu method was used with minor modifications. A total of 20 µl of the extract was combined with 100 µl of Folin–Ciocalteu reagent (1:10 v/v) and 80 µl of 7.5% sodium carbonate. The mixture was shaken for 30 s and allowed to stand for 2 h at room temperature in darkness. Standards (gallic acid) and blanks (methanol) were prepared and processed using the same method as for SWE. The absorbance was measured at 765 nm using a microtiter plate (SPECTROstar Nano, BMG Labtech Germany). Gallic acid was used as standard, and blanks (methanol) were prepared following the same method as for seaweed samples. Results were expressed as mg gallic acid equivalents/g dry extract.

##### 2.2.1.6 Total flavonoid content

Seaweed ethanolic extracts were obtained previously as described in the previous section. Total flavonoid content was determined as described by Chang et al. (2002). Briefly, 15 µl of the sample was mixed with 307 µl of 10% aluminium chloride, 307 µl of 1M potassium acetate, and 200 µl of Milli-Q water. After incubation for 30 min in the dark at room temperature, the absorbance was measured at 510 nm (SPECTROstar Nano, BMG Labtech Germany), using quercetin as standard.

##### 2.2.1.7 Total condensed tannin

The total condensed tannin content was performed by the vanillin–HCl method (Cox et al. (2010). Seaweed methanol extract (25 µl), obtained as in *Section 2.2.1.5*, was mixed with 150 µl of vanillin 4% and 75 µl of HCl concentrated. The solution was shaken and left at room temperature for 20 min of protected light. The absorbance of samples, standards ((+) catechin), and blanks (methanol) were recorded using a microtiter plate (SPECTROstar Nano, BMG Labtech Germany) at 500 nm.

##### 2.2.1.8 Total carotenoid content

Carotenoids were determined according to the methodology of Kumar et al. (2009), with slight modifications. Briefly, 1 g of powdered samples was incubated with 10 ml of 80% acetone for 90 min at 20°C. Then, the mixture was centrifuged for 15 min at 3,000 rpm and filtered using a filter paper (Whatman No. 1). A standard curve was prepared using β-carotene, and results were obtained by measuring the absorbance at 480 nm (SPECTROstar Nano, BMG Labtech Germany).

##### 2.2.1.9 Total antioxidant capacity

Total antioxidant capacity (TAC) was assessed according to a previous method (Costa et al., 2011). Seaweed methanolic extract (100 µl), obtained as previously described, was mixed with 1 ml of a solution containing 0.6 M sulphuric acid, 28 mM sodium phosphate dibasic and 4 mM ammonium molybdate (4:2:4 v/v/v). The mixture was incubated for 90 min at 95°C and cooled at room temperature. Then, the absorbance of standards (ascorbic acid), blanks (methanol), and samples were measured at 695 nm (SPECTROstar Nano, BMG Labtech Germany).

##### 2.2.1.10 DPPH radical scavenging activity

The DPPH radical scavenging activity was determined using the method of Zhang et al. (2008) and Yang et al. (2008). Seaweed methanol extract (100 µl) and 0.16 mM 2,2-diphenyl-1-picrylhydrazyl hydrate (DPPH) (100 µl) in methanol were mixed. The mixture was shaken for 30 s and left in darkness at room temperature for 30 min. The absorbance was read in a microtiter plate (SPECTROstar Nano, BMG Labtech Germany) at 517 nm. The scavenging effect was calculated as an inhibition percentage using **Eq. (1)** as follows:


$$DPPH\;inhibition(\%)=\left(\frac{A_{c\;}-A_{s}}{A_{c}}\right)\times \;100$$

where A_c_ is the absorbance of the control (100 µl of methanol solvent with 100 µl of the DPPH solution), and A_s_ is the absorbance of the sample.

#### 2.2.2 *In vitro* antifungal activity assays

Previously, the products under study were tested to verify that they were free of microorganisms. For that purpose, the products were prepared at recommended dose by the manufacturer, and 500 µl was incubated on Petri dishes with a general culture medium, potato dextrose agar (PDA), to check the presence of any kind of microorganism. All the samples were done in triplicate. They were incubated at 26°C in the dark in a growth chamber for a week.

Pathogen fungi were isolated from the selected rotten fruits: *Penicillium digitatum* from orange, *Botrytis cinerea* from strawberry, blueberry, and tomato in PDA. Petri dishes were incubated in a growth chamber at 26°C in the dark. Once grown, 5-mm fungal plugs were inoculated in PDA plates, and the correspondent product was poured at a recommended dose by manufacturer, all in triplicate. They were grown in the same conditions for 22 days. The growth inhibition halo was evaluated.

### 2.3 Statistical analysis

IBM SPSS Statistics 26.0 software (SPSS Inc., Chicago, IL, USA) was used for multivariate analysis of covariance (MANCOVA), principal component analysis (PCA), hierarchical cluster analysis (HCA), and artificial neural network analysis (ANN).

## 3 Results

### 3.1 Chemical composition

The results of the analysis of characteristic and bioactive compounds, including the mean (and standard deviation), minimum, and maximum values of the products evaluated, are shown in **Table 2**. The selected products presented a wide range of pH from 3.9 to 10.7. This variety is mainly due to the extraction method used and the algae percentage in the product. The EC provides the value of the soluble salts present in the SWEs. The strong differences observed among the products can be attributed to the way in which the algae are extracted, e.g., alkaline media provide high concentrations of potassium, and/or the area where they have been collected (mainly *AN* grows in the Atlantic Ocean and *EM* in South Africa). It is recommended that a fertilizer has a low EC value to facilitate fertilization and avoid crop phytotoxicity. The variations in AA, Man-ol, and Lam were attributed to intrinsic botanical differences of the species. It was observed that *AN* products presented higher contents for these three components than *EM*. These results agreed with those reported in previous studies and can also be attributed to the different growing conditions of the source material and possible variations in the processing methods (Pereira et al., 2020; Valverde et al., 2022). Sugar profile was similar for both species, following the trend: glucose≃ glucuronic acid>sucrose≃ fucose>xylose. Although the concentration of sugars was higher for *AN*, all the products presented a variety of sugars that will facilitate the assimilation of nutrients and their transport by the plant, as they reduce the osmotic pressure, thus improving vegetative development. The mineral composition for *AN* product (**Table 2**) followed the trend, K>Mg>Ca>Al>P>Fe>Mn>Zn>Cu, whereas *EM* followed the trend, K>Mg>Ca>P>Al>Fe>Zn>Mn>Cu. Potassium, Mg, and Ca were the most abundant plant macronutrients in the studied products, highlighting that the higher concentrations of K may be due to the accumulation in the tissues of the algae and extraction methodology employed. Variations in microelements are attributed to the species of algae, the season in which they were collected, and the algae percentage of the product. Heavy metals were analyzed because seaweeds are known to accumulate pollutants. The results revealed the presence of Cr and Ni in most of the products, which can affect both the nutrition of the plants and their productive quality. *A. nodosum* products presented the higher concentrations (Ni, 0.15 ± 0.002 mg/kg; Cr, 0.19 ± 0.001 mg/kg) although within the established limits by European Commission under Regulation (UE) 2019/1009 about fertilizer products (Ni= 50 mg/kg and Cr=2 mg/kg dry matter). These results are in relation to those previously reported, indicating that *AN* is used as a biomonitor of metal concentration in coastal habitats (Morrison et al., 2008). The results of the hormonal analysis were conditioned by the instrumental analysis technique used (HPLC-DAD) because these analytes are expected in low concentrations. Despite this, the most remarkable results, as expected, were for indole-3-acetic acid (IAA), the major hormone. It is the main auxin in plants and controls several physiological processes such as cell elongation and division. It was detected in products of both species; however, it was not detected in the solid formulations. This fact has not been previously reported in any studies, which suggests that the type of formulation may influence in some way the final hormonal composition of the product. The analysis of bioactive compounds revealed that the concentration of TFC (274 ± 2.0 g/kg) was higher than that of TPC (107 ± 1.9 g/kg) in all the products tested, *AN* being the products with the highest concentration. Notable concentrations of TCC (14.6 ± 0.87 g/kg), associated with the characteristic brownish color, were also detected with the same trend described above. Most of the samples presented tannins although at very low concentrations. It should be noted that the presence and abundance of these compounds depend on the species, the habitat, the seasonal influence, the stage of the life cycle, the environmental conditions, and the extraction method. All the products tested presented high concentrations of bioactive components as described in previous studies (Agregán et al., 2017; Kocira et al., 2018; Habeebullah et al., 2021). The presence of these compounds will benefit the productive development of the plant and the quality of the fruit, providing them high added value. The antioxidant activity was evaluated by TAC and DPPH assays. According to the parameters studied previously, it was observed that the TAC was higher for *AN* (24.4 ± 0.32 g/kg) than for *EM* (0.84 ± 0.32 g/kg). These results are related to the presence of antioxidant compounds, which agree with the results obtained previously. DPPH assay has been used extensively as a free radical to evaluate the antioxidant potentials in plant extracts (Duan et al., 2006; Farasat et al., 2014). SWEs contain numerous antioxidant molecules that are able to reduce the DPPH free radicals by attacking the molecules to add hydrogen atom to it or donate an electron to it, providing a color change from purple to yellow in the DPPH solution (Devi et al., 2011). The average values for scavenging DPPH radical were always higher than 40% for most products, with a trend similar to that observed in TAC assay. Previous studies reported that brown seaweeds are rich in bioactive compounds compared with red and green seaweeds, so %DPPH reduction is greater (Cox et al., 2010; Leelavathi & Prasad, 2014).

**Table 2:** Summary of the compounds analyzed in seaweed extracts under study.

Variables		Min.	Max.	Mean.	SD
pH (1:5)^a^, (1:25)^b^	pH	3.79	10.7	7.38	0.04
Electric conductivity (mS cm^−1^)	EC	2.17	42.7	22.4	0.1
***Characteristic compounds***					
Alginic acid (%)	AA	0.61	18.0	9.21	0.2
Mannitol (%)	Man-ol	<LOD^c^	5.84	2.92	0.05
Laminarin (%)	Lam	<LOD^c^	4.73	2.37	0.2
Potassium (g/kg)	K	5.04	180	92.5	1.5
Calcium (g/kg)	Ca	0.40	11.6	6.00	0.5
Magnesium (g/kg)	Mg	0.11	10.3	5.13	0.2
Aluminum (g/kg)	Al	0.20	2.10	1.15	0.05
Phosphorus (g/kg)	P	0.16	2.82	1.50	0.1
Iron (g/kg)	Fe	0.046	1.26	0.65	0.1
Copper (g/kg)	Cu	<LOD^c^	0.50	0.25	0.01
Manganase (g/kg)	Mn	<LOD^c^	0.75	0.38	0.05
Zinc (g/kg)	Zn	0.033	1.51	0.77	0.07
Chromium (mg/kg)	Cr	<LOD^c^	0.19	0.096	0.001
Nickel (mg/kg)	Ni	<LOD^c^	0.15	0.045	0.002
Sucrose (mg/kg)		<LOD^c^	70.0	34.9	0.84
Glucuronic acid (g/kg)		<LOD^c^	81.3	40.6	1.2
Glucose (g/kg)		5.10	31.1	18.1	0.7
Xylose (g/kg)		<LOD^c^	32.6	16.3	0.3
Rhamnose (g/kg)		<LOD^c^	5.16	2.58	0.4
Arabinose (g/kg)		<LOD^c^	5.40	2.69	0.2
Fucose (g/kg)		<LOD^c^	69.6	34.8	1.9
∑%Sugars		0.20	14.5	7.30	0.2
Indole-3-acetic acid (g/kg)	IAA	<LOD^c^	16.6	8.27	0.8
Salicylic acid (mg/kg)	SA	<LOD^c^	54.3	27.1	2.1
***Bioactive compounds***					
Total phenol content (g/kg)	TPC	1.80	107	54.6	1.9
Total flavonoid content (g/kg)	TFC	1.90	274	138	2.0
Total tannin condensed content (µg/kg)	TTC	0.01	0.65	0.30	0.05
Total carotenoid content (g/kg)	TCC	0.30	14.6	7.50	0.9
Total antioxidant capacity (g/kg)	TAC	0.82	24.2	12.5	0.3
DPPH radical scavenging activity (%)	DPPH	0.00	87.3	43.7	1.3

Mean (n=20) values.

a pH dilution (1:5) for liquid products.

b pH dilution (1:25) for solid products.

c The measurement was under the detection limit (LOD).

### 3.2 Antifungal activity

All products under study were evaluated to elucidate if they could have any effect on the most common pathogen fungi of orange, strawberry, blueberry, and tomato. The presence of inhibition haloes was considered positive, as the products used in crops could inhibit the growth of these undesirable pathogens. Some of the tested products inhibited the fungal growth, as is shown in **Table 3** with a (+) sign at 48 h, although the cultures were incubated for 22 days to know if the effect persisted. The initial microbiological test showed that at recommended doses, products 8 and 12 promoted in themselves the growth of some kind of microorganisms. The plates with product 8 seemed to grow fungi; meanwhile, product 12 showed a bacterial growth, but specialized research was required to properly describe them (see **Supplementary Figure S1**). Thus, their use might not be recommended, as the effect on soil microbiota and on fruits is not known and should be evaluated in further research. It should be noted that both products correspond to *AN* with low algae percentage. The results showed the products that inhibited both fungal growth at 48 h and continued with the effect for 22 days. *Botrytis cinerea* in tomato was inhibited in 65% (4,6,8–14,16–20) of the products studied, while in strawberry and blueberry, there were fewer products that presented action, 25% (6,10–13) and 35% (4,6,8–9, 16–17,20), respectively. Noteworthy, most of the products that showed inhibition in strawberry corresponded to *EM*. *Penicillium digitatum* was inhibited in 25% of the products studied (5,8–9,13,17). The products that inhibited the growth in more fruits were the following: product 6 against *B. cinerea* in all of the fruits studied; products 8, 9, and 17 showed a positive effect on tomato, blueberry, and orange; and product 13 against pathogens in tomato, blueberry, and orange. In **Supplementary Figure S2**, the Petri dishes of tomato and strawberry with product 13 (*EM*, 100%) are shown; the inhibition halo can be clearly seen especially in strawberry at 48 h (**Supplementary Figure S2E**), and the effect remains for 22 days (**Supplementary Figure S2F**).

**Table 3:** Results of antifungal activity *in vitro* after 48 h of application of the SWEs.

Sample code	Tomato	Blueberry	Strawberry	Orange
1	−	+	−	−
2	−	−	−	−
3	−	−	−	−
4	+	+	−	−
5	−	−	−	+
6	+	+	+	−
7	−	−	−	−
8	+	+	−	+
9	+	+	−	+
10	+	−	+	−
11	+	−	+	−
12	+	−	+	−
13	+	−	+	+
14	+	−	−	−
15	−	−	−	−
15	+	+	−	−
17	+	+	−	+
18	+	−	−	−
19	+	−	−	−
20	−	+	−	−

Symbol (-) represented the absence of effect of SWEs on the studied pathogens. Symbol (+) was associated with the positive effect of SWEs with the inhibition haloes on the studied pathogens.

These facts indicate that products of both species (*AN* and *EM*) showed inhibition halo and products of different percentage and type of formulation. **Table 4** shows the most relevant previous works where the efficacy of SWE against different pathogens was evaluated. Only one previous study was found in which the effectiveness of *EM* was evaluated (Righini et al., 2020). In this research, *EM* did not present antifungal activity; it should be noted that both the pathogen and the fruit studied were different (*P. xanthii* in cucumber). Our study showed promising results for *EM* extracts especially in strawberry. Furthermore, previous studies also found a positive activity of *AN* against the pathogen *B. cinerea* in carrot, cucumber, and pear (Jayaraj et al., 2008; Jayaraj et al., 2011; Lutz et al., 2022) and similar results in *L. digitata* (common brown seaweed used in agriculture) in strawberry (De Corato et al., 2017). The antifungal effect of those products was related to polysaccharide content, carbohydrate content, or bioactive molecules of the algae such as polyphenols or pigments like carotene (see **Table 4**). In these studies, the SWE used presented 12%–18% alginic acid, 5%–6% Man-ol, and 10%–15% carbohydrate content, comparable to the solid products of this study (17–20), which showed activity against *B. cinerea* in different fruits. *Penicillium digitatum* was evaluated to a lesser extent; only two recent studies were found in brown algae. One of them evaluated the species *L. digitata* in citrus lemon (De Corato et al., 2017), and its positive effect was related to the content of polysaccharides (23%) and TPC (2.3%). In general, this content was higher than in the products of this study that presented positive activity against *P. digitatum*, but a relationship between two products was found: product 17 presented 19% polysaccharides content and product 5 a 2.5% TPC. Lahbib et al. (2022) related the fungicidal activity with TPC (14.26 mg/g) and TFC (9.16 mg/g). The products of this study present TPC values between 7–17 mg/g but much higher for TFC (30–240 mg/g). All these suggest that the antifungal activity may be due to the combined action of different compounds. Although it is true that previous works have evaluated other types of algae (red and green), brown algae are the ones that presented the highest fungicidal capacity (De Corato et al., 2017; Righini et al., 2020; Lahbib et al., 2022). This study evaluated for the first time the action of *AN* and *EM* against *P. digitatum*. Promising results were found for tomato (65% of the products showed positive action) and *EM* in strawberry. It should be noted that no previous studies on blueberries were found. In addition, 20 SWEs were evaluated compared to 5 in other studies, being the maximum number of products studied in previous research and providing a much more detailed information on the chemical composition. Moreover, this study lasted for 22 days to check the long-term fungicidal effect, while previously reported studies did not indicate this effect. However, in those pathogens, more research should be done, since the application of algae extract to fertilize the crops could have a positive effect on inhibiting pathogens, providing added value to these products. These promising results indicate that these products could be an alternative to use in to treat fruits and vegetables postharvest to minimize the damage caused by the attack of microorganisms.

**Table 4:** Antifungal activity of SWEs reported in published studies.

Algae	Species	SWE (n°)	SWE composition	Pathogen	Fruit/vegetables	*In vitro*	*In vivo*	Compounds involved	Ref.
Brown	*AN*	1	PS and CH	*Alternaria radicina*, *B. cinerea*	Carrot	No	Yes	CH and laminarin	Jayaraj et al., 2008
Brown	*AN*	1	PS and CH	*Alternaria cucumerinum*, *Fusarium oxysporum, B. cinerea*	Cucumber	No	Yes	CH, laminarin and carragen	Jayaraj et al., 2011
Brown and red	*L. digitata, U. pinnatifida, P. umbilicalis, E. denticulatum, G. pusillum*	5	Lipids, fatty acids, PS, and TPC	*B. cinerea, Monilinia laxa, P. digitatum*	Strawberry, peach and citrus lemon	Yes	Yes	Phenolic compounds, PS and lipids	De Corato et al., 2017
Brown and red	*EM* and *J. adhaerens*	2	Proteins, chlorophylls, carotenoids and antioxidant activity	*Podosphaera xanthii*	Cucumber	Yes	No	Proteins and carotenes	Righini et al., 2020
Red	*Gracilaropsis persica*	1	Bioactive compounds	*B. cinerea*, *Pyricularia oryzae, Aspergillus niger*, and *Penicillium. expansum*	Fungi collection (laboratory)	Yes	No	Phenolic compounds and tannin	Pourakbar et al., 2021
Brown	*AN*	1	NS	*B. cinerea*, *A. alternata*, and *Alternaria* spp.	Pear	Yes	Yes	NS	Lutz et al., 2022
Brown, red and green	*U. fasciata, (Rhodophyceae) B. bifurcata, (Chlorophyceae)*	4	TPC, TFC, and antioxidant activity	*P.digitatum*, *Penicillium expansum*, and *Penicillium italicum*	Fungi collection (laboratory)	No	Yes	Phenolic compounds and fatty acids	Lahbib et al., 2022
Brown	*AN* and *EM*	20	PS, mineral profile, CH content, hormonal composition, TPC, TFC, TCC, TTC, and antioxidant activity	*B. cinerea* and *P. digitatum*	Tomato, blueberry, strawberry and orange	Yes	No	PS, CH content and bioactive compounds	Present study

PS, polysaccharide content; CH, carbohydrate content; NS, not specified.

These findings revealed that market SWEs have a great variety in their composition, thus justifying this study and the need to continue with this line of research carrying out tests *in vivo*.

### 3.3 Chemometric analysis

#### 3.3.1 Multivariate generalized linear model

MANCOVA analysis was used to evaluate the effect of algae species and algae percentage on the final composition of the products studied. It was conducted to test the influence of several independent variables in structural (AA, Man-ol, Lam, metal profile, IAA, SA, and sugar content) and bioactive compounds (TPC, TFC, TCC, TTC, TAC, and DPPH) of products studied. In order to make a preliminary determination of algae species, the percentage of SWE in the product and the extraction process were used as fixed factor and the formulation type as covariate. To evaluate the statistical significance of the model, Wilks’s lambda, F-value, and p-values for main and interaction effects were employed. Levene’s test of equality of error variances was given for all measured parameters. The level of significance was set at p<0.05. The results showed that the strongest explanatory variables were the percentage of SWE in the product (Wilks’s λ=0.02, F=4.045, p=0.028) and algae species (Wilks’s λ=0.01, F=190.9, p=0.047); meanwhile, the extraction process (Wilks’s λ=0.01, F=3.080, p=0.164) had no significant effect, so we omitted this variable in the final MANCOVA model. The formulation type did not interact with fixed factors and had no significant effect (Wilks’s λ=0.02, F=81.98, p=0.085), indicating that this covariate was selected correctly. The reported tests showed that for these variables—species and SWE percentage—the null hypothesis is rejected, so they significantly influence on the final composition of the products. These findings contribute to understanding the role of variables related to the production process on the final composition of SWE.

#### 3.3.2 Principal component analysis

**Figure 1:**
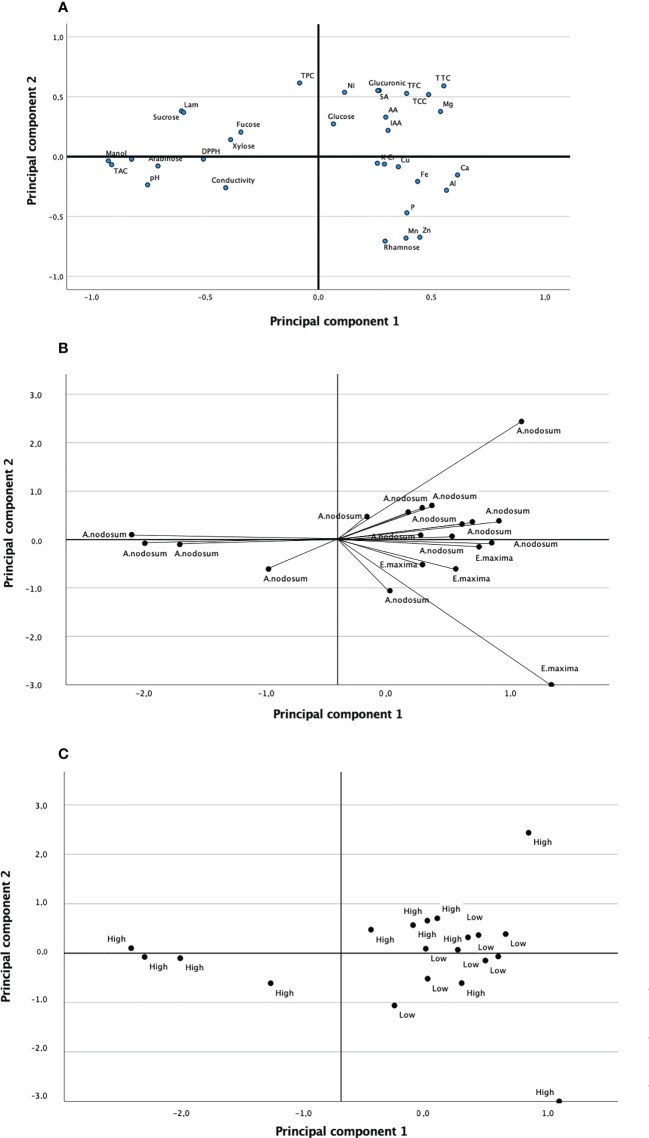
**(A)** Principal components plot in rotated space of variances of seaweed extracts based on chemical composition; **(B)** PCA based on algae species (*AN* and *EM*); **(C)** PCA based on algae percentage in products studied (low and high).

PCA was made using the raw data obtained from the composition analysis of SWE. To examine the suitability of these data, the Kaiser–Meyer–Olkin (KMO) test was performed. The KMO test measures the sampling adequacy and indicates the proportion of variance. The KMO result was 0.82 (≥0.5), indicating that PCA could be useful in providing significant reductions in the dimensionality of the raw data. Major components were extracted with eigenvalues >1.0. In this model, PC1 explains 35% of the accumulative variance; PC2, 60%; PC3, 69%; PC4, 75%; PC5, 84%; PC6, 87%; PC7, 91%; PC8, 92%; and PC9, 99%. The component plot rotated space generated from the first two PCs explained the variations in the seaweed products in relation to their composition (**Figure 1A**). PC1 had a medium positive loading (loading values, 0.50–0.70) of Ca, Mg, Al, TCC, and TTC and strong negative loading (loading values, 0.7–1.00) for Man-ol, TAC, and pH. Most of the samples showed a positive relation with PC1. On the other hand, a high negative relationship was observed corresponding to the solid formulations (see in **Figure 1B**) due to their low concentration of Ca, Mg, and Al and high levels of Man-ol, pH, and TAC. PC2 showed a medium positive loading (loading value, 0.50–0.70) of TPC, Ni, glucuronic acid, SA, TFC, TCC, and TTC and a medium negative loading of rhamnose, Mn, and Zn. As can be seen in **Figure 1B**, the product that presented the greatest negative relationship corresponded to sample number 13 and product 14 and showed a positive relationship with PC2. Product 13 is related to species EM and products with the highest algae percentage content. Hence, it has the lowest values for these variables and the highest value for rhamnose, which is specific for *EM*. Product 14 was related to *AN*, had 100% algae percentage, and had the highest values for these variables, especially Ni (0.15 ± 0.002 mg/kg) and no rhamnose levels. *AN*, hence, is found at a higher relationship with PC2 than EM. Overall, these findings are in accordance with the MANCOVA and concluded that composition profiling was influenced by species and percentage of seaweed in the formulation. In this way, it can be concluded that PCA satisfactorily highlighted the differences between the products based on the species and percentage of algae (**Figure 1C**).

#### 3.3.3 Hierarchical cluster analysis

**Figure 2:**
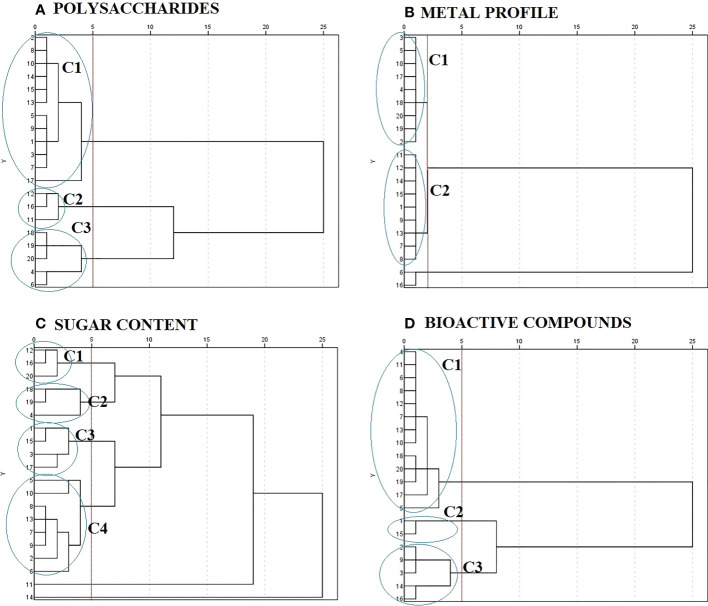
Dendrogram of hierarchical cluster analysis of seaweed extracts based on: **(A)** polysaccharides composition, **(B)** metal profile, **(C)** sugar content, and **(D)** bioactive compounds.

HCA allows to classify the products studied according to their similarities in chemical composition. It was performed using Ward’s method with squared Euclidean distances to examine station similarities. Ward’s method uses ANOVA to calculate the distance between groups to minimize the sum of squares of two possible groups at each step. **Figure 2A** shows the dendrogram of the products based on the analysis of polysaccharide’s composition. Three well-defined main clusters were observed. Cluster 1 was clearly discernible and constituted by low algae percentage products mainly obtained through cold extraction and acid media. Clusters 2 and 3 were explained by algae percentage of products (100%) and the type of formulation; all solid products were grouped in these clusters and represented the products with the high polysaccharide content, between 8% and 12%. HCA results for the mineral profile is shown in **Figure 2B**. Two clusters with great similarities were observed, which is in good agreement with the previously described results. Most of the products follow the same trend in the mineral profile with the exception of products 6 and 16. These products showed large Euclidean distance, which indicated dissimilarity between the other products. **Figure 2C** represents the dendrogram of the sugar content. Clusters 1 and 2 are established by the products with the highest sugar content. They correspond to *AN* products, and most of them are in solid formulation. Clusters 3 and 4 are formed by the samples that have 3%–5% sugar content. The products with the highest sucrose content were grouped in cluster 3, while cluster 4 was formed by those with the highest glucose content. All the products of EM under the study were in cluster 4. Sample numbers 11 and 14 were the most different and had more than 15% sugar content. **Figure 2D** shows **3** main clusters for bioactive compounds and antioxidant activity. Cluster 1 grouped 13 products with the highest DPPH percentage (products 7, 8, 10, 11). This cluster was defined by high percentage SWE products, both *AN* and EM, and all solid products were in this group. Cluster 2 was composed of the products with the highest TPC and cluster 3 with those that had the highest TFC. Both clusters presented similarities in terms of being formed by the products that correspond to mixtures (low algae percentage). This fact suggests that the products are mixed with some components that could enhance the presence of phenolic compounds. HCA has given a clear view of the composition pattern of SWE of the respective species and algae percentage. These results are in very good agreement with those observed in MANCOVA and PCA.

On the other hand, it is well-known that antifungal activity is related to the presence of multiple compounds, and HCA can be a useful tool to relate it. The products that presented action against *P. digitatum* (5, 8, 9, 13, and 17) were grouped in the same polysaccharide cluster (cluster 1), and for carbohydrates, four of them were grouped in cluster 4 and the other one in cluster 3. The characteristics of these clusters have been discussed previously; however, it should be noted that sucrose was not detected in these products. Sucrose is used by pathogens as a carbon source; possibly, its absence has inhibited their growth. In the case of tomato, the products of interest were grouped in cluster 1 (4, 6, 8, 10–13, 17–20) and cluster 3 (9, 14, 16) for bioactive components. The products that presented action on strawberry (6,10–13) were grouped in cluster 1 of bioactive components and that on blueberry were grouped in cluster 1 (4, 6, 8, 17, 20) and cluster 3 (9, 16). As can be seen, the fungicidal effect on *B. cinerea* was associated with polyphenolic compounds as described in other works (**Table 4**), while for *P. digatum*, it was associated with polysaccharide and carbohydrate content.

#### 3.3.4 Artificial neural network analysis

ANN have gained popularity in recent years for modeling methodologies for food quality and food processing (Bhagya Raj and Kshirod, 2022). This methodology has numerous advantages such as flexibility, mapping ability, and high-speed information processing ability, and the ability to solve non-linear complex analysis even if the exact nature of the non-linear relationship between the input and output neurons is unknown (Abiodun et al., 2018). A multilayer perceptron (MLP) based on back propagation (BP) was applied for modeling the composition of the seaweed products. The best model analysis conditions were as follows: the activation function and the number of hidden layers were hyperbolic tangent and four layers, respectively. The output layer activation function was Softmax, and the training type was batch training. Under this condition, the correct rate of model training and testing was 97.3% and 91.7%, respectively. The results showed that the SWE can be distinguished according to the components analyzed. Under this model, the components that made the greatest contribution to classification and their normalized importance were Zn (100%), EC (95%), K (91%), glucuronic acid (86%), TCC (77%), and Mn (72%).

## 4 Conclusions

This study has contributed to the knowledge about commercial SWEs used in agriculture. The composition of 20 commercial products with relevant characteristics and their antifungal potential against the usual fungi of certain fruits were analyzed. These results revealed important information about them and can contribute to improved labeling, whose problem is latent. Chemometric techniques were applied using linear and non-linear models in order to establish how the variables related to the production of the extracts influence the chemical composition of the products. MANCOVA allowed to determine the variable species and algae percentage in products as the most influential (p-value and Wilk’s lambda test). Principal component analysis and hierarchical cluster analysis discriminated the dependent variables according to species and algae percentage. PCA revealed that rhamnose was a characteristic in *E. maxima* with 100% seaweed in its composition; this parameter could be used to control fraud in these products. In addition, a characteristic composition was observed in the solid products, defined by low concentrations of Ca, Mg, and Al and high concentrations of mannitol, total antioxidant content, and high pH values. HCA showed that the highest concentration of bioactive components was grouped in the same cluster corresponding to low algae percentage. This fact suggests that some component has been added to the products. In addition, HCA was used to relate the antifungal capacity of SWE to their composition. These results revealed that the fungal capacity against *B. cinerea* was related to the bioactive components, while that against *P. digitatum* was related to the polysaccharides and carbohydrates content. Moreover, ANN allowed to establish the influence of other variables (K, glucuronic acid, laminarin, and total carotenoid content) that were not revealed by the linear methods. The overall results could help to better understand the mode of action of these products through their composition and the importance of chemometric techniques to discern the most significant variables in the production process and their relationship with their fungal capacity. Future research on other products available on the market is recommended to deepen the investigation related to postharvest treatments due to their action against common pathogens in fruits.

## Data availability statement

The original contributions presented in the study are included in the article/**Supplementary Material**. Further inquiries can be directed to the corresponding author.

## Author contributions

SV, JJL and LHA designed the experiments. SV and PLW performed the analysis of chemical composition of SWEs. SV, PLW and BM performed the microbiological assays. SV and PLW acquiered and analyzed of data; BM, JJL and LHA hepled perform the analysis of data with constructive disscussions. SV, PLW and LHA wrote the manuscript. All authors revised the manuscript and approved the submitted version.
