# Supplementary material for: Comparative study of the chemical composition and antifungal activity of commercial brown seaweed extracts
Source: Front Plant Sci. 2022 Dec 13;13:1017925. doi: 10.3389/fpls.2022.1017925 (PMC9792768; doi:10.3389/fpls.2022.1017925)
Supplement: Supplementary file 1 [file DataSheet_1.docx]

Supplementary Material

**Figure S1.-** Petri dishes which showed growth of microorganism after application of products number 8 and 12.

**
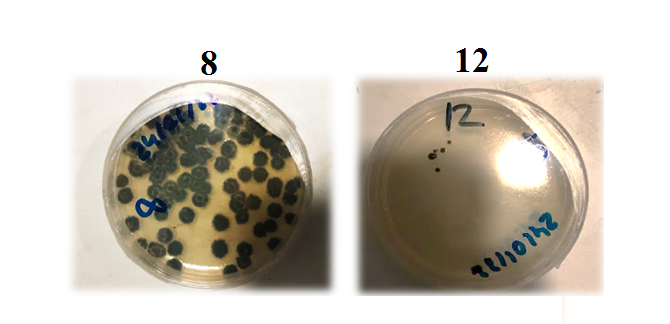
**

**Figure S2.-** Petri dishes of the pathogen fungi tested with product 13 in Tomato: **A)** control, **B**) at 48 h; and **C**) at 22 days. Strawberry: **D)** control, **E)** at 48 h and **F)** at 22 days.

**
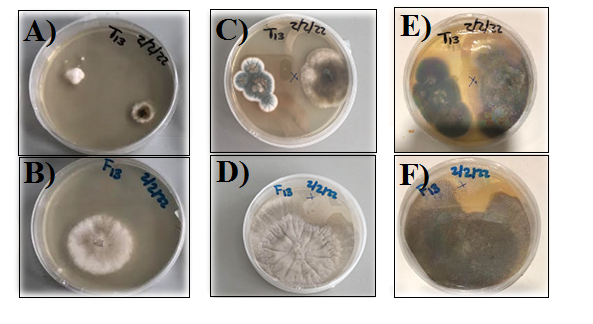
**

**Table S1.** Optimal conditions for ICP-OES with selected wavelenghts and internal standard (IS).

| **Parameters** | **Value** |  |
| --- | --- | --- |
| **Forward power (W)** | 1150 |  |
| **Ar gas flow rated (L/min)** |  |  |
| **Nebulizer** | 0.5 |  |
| **Coolant** | 12.5 |  |
| **Auxiliary** | 0.5 |  |
| **Nebulizer gas pressure (bar)** | 1.92 |  |
| **Replicates** | 3 |  |
| **Uptake time (s)** | 50 |  |
| **Pump speed (rpm)** | 45 |  |
| **Analysed analyte** | Wavelenghts (nm) | |
|  | 315.887Ca | 213.856Zn |
|  | 279.553Mg | 267.716Cr |
|  | 766.490K  177.495P  167.079Al | 221.647Ni  189.042As  214.438Cd |
|  | 259.940Fe | 220.353Pb |
|  | 324.754Cu  257.853Mn |  |
| **IS** | 371.030Y |  |

**Table S2.** MANCOVA analysis of seaweed products.

|  | **Hypothesis df** | **df error** | **F-value** | **p-value** | **Wilks’ λ** |
| --- | --- | --- | --- | --- | --- |
| Algae specie | 11 | 1 | 25.386 | 0.041 | 0.01 |
| Algae percentage | 55 | 8.216 | 3.821 | 0.023 | 0.04 |
| Algae specie*algae percentage | 11 | 1 | 7.974 | 0.275 | 0.11 |

^A^ df is degree of freedom

.
